# Supplementary material for: Neural network approaches for solving Schrödinger equation in arbitrary quantum wells
Source: Sci Rep. 2022 Feb 15;12:2535. doi: 10.1038/s41598-022-06442-x (PMC8847422; doi:10.1038/s41598-022-06442-x)
Supplement: Supplementary file 1 — Supplementary Information. [file 41598_2022_6442_MOESM1_ESM.docx]

**Appendix**

1. For each potential function in DS1, a different number $n$ of angular points between 3 and 13 is randomly generated, using a normal distribution with mean 3 and standard deviation 5. Intuitively, this number will be related to the intricacy of the potential spatial variation and is not allowed to be too large, since practical QWs are rather considered. A preliminary discretization $\left\{ \eta_{l} \right\}_{1\leq l\leq n}$ of the well interval $\left[ -1,1 \right]$ is randomly generated using an uniform distribution, and overridden so that $\eta_{1}=-1$ and $\eta_{n}=1$. An upper envelope discrete function $\left\{ U_{l} \right\}_{1\leq l\leq n}$ is empirically defined in such a way as to ensure the existence of at least one bound energy level in the QW: $U_{l}=min\left\{ \frac{abs\left( \eta_{l} \right)+1}{2},\frac{7}{10} \right\}$. The discrete potential values $\left\{ u_{l} \right\}_{1\leq l\leq n}$ corresponding to $\left\{ \eta_{l} \right\}_{1\leq l\leq n}$ are then randomly chosen in the interval $\left[ 0,U_{l} \right]$ by using a uniform distribution, and overridden so that $u_{1}=u_{n}=1$. Finally, the potential discrete function $\left\{ \xi_{i} \right\}_{1\leq i\leq M}\to\left\{ v_{i} \right\}_{1\leq i\leq M}$ is obtained by interpolating the set of preliminary points $\left\{ \left( \eta_{l},u_{l} \right) \right\}_{1\leq l\leq n}$ in the discrete set $\Xi_{\mathrm{in}}$ and forcing all the values outside the well to be 1. The interpolation is arbitrarily either linear or piecewise cubic Hermite polynomial. In Fig. A1 some random examples are given.


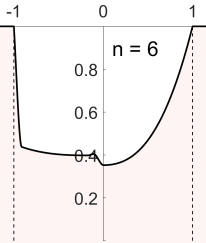

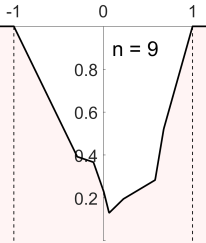

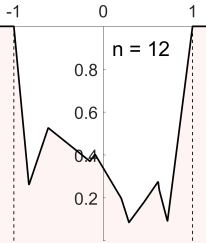

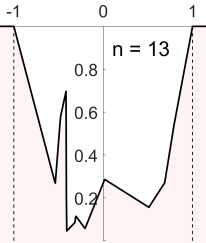

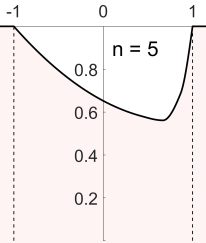

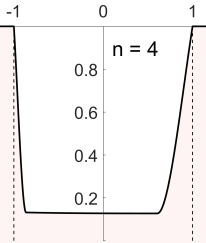

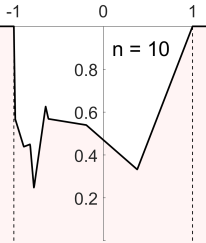

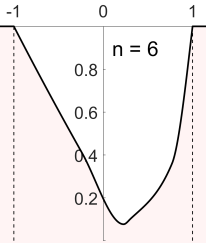

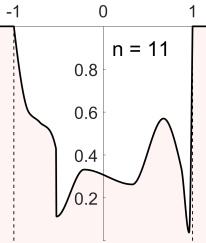

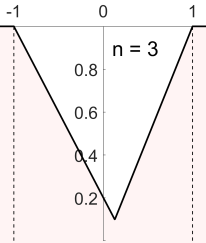

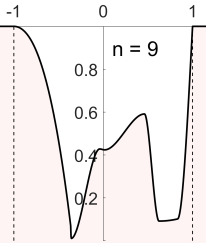

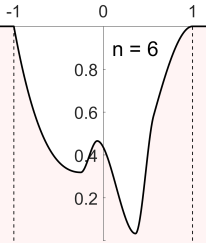

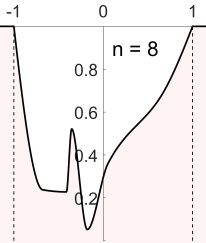

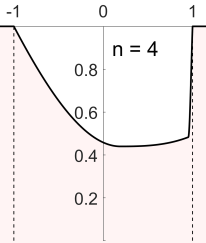

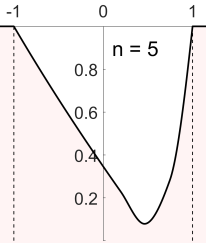

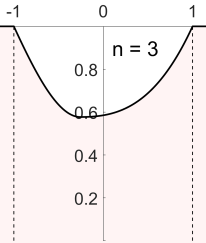

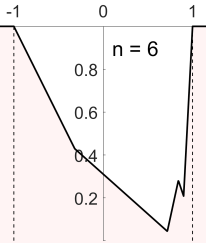

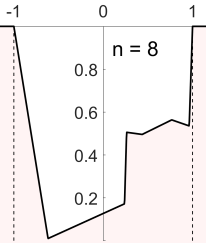


Fig. A1. Several random DS1 potentials.

1. The potentials in DS2 are prepared similarly to those in DS1, except for the final interpolation, which is of the type nearest neighbor (see Fig. A2).


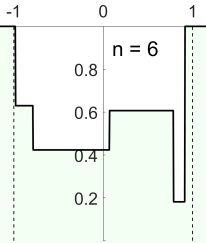

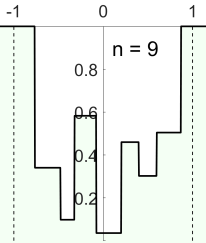

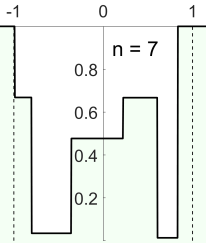

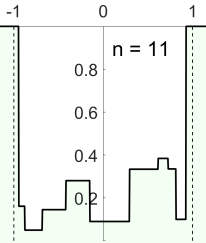

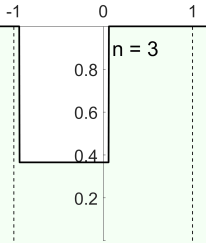

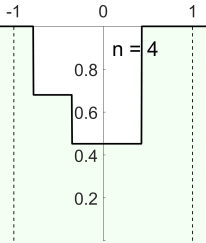

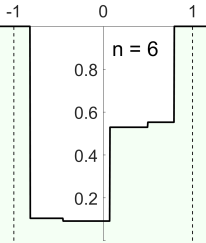

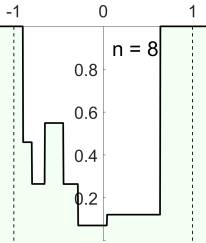

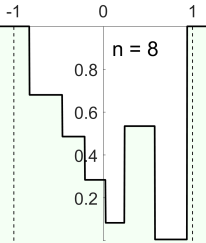

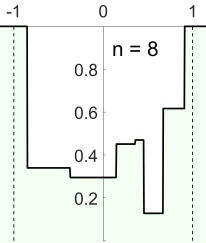

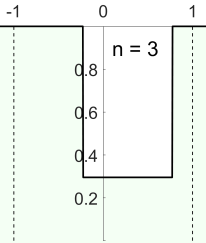

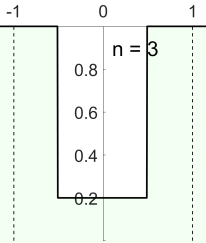

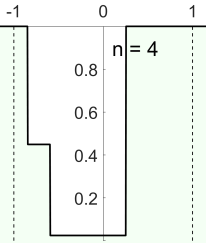

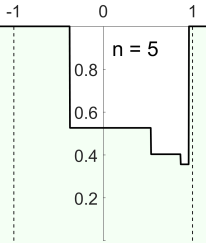

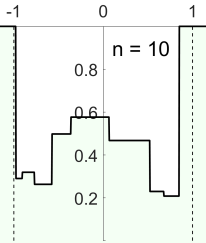

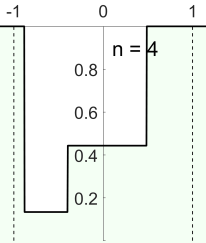

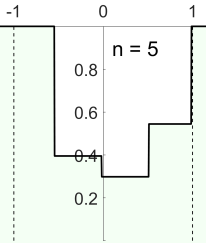

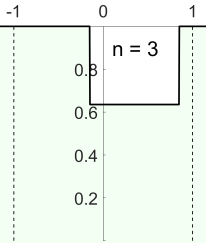


Fig. A2. Several random DS2 potentials.

1. DS3 potentials are based on Fourier synthesis. A random number $n$ of Fourier terms is prepared for each sample following the procedure described for DS1. Fourier coefficients $\left\{ a_{l} \right\}_{1\leq l\leq n}$ and $\left\{ b_{l} \right\}_{1\leq l\leq n}$ are generated randomly in the interval $\left[ -1,1 \right]$ using a uniform distribution. In a first step, the sums of $n$ harmonic terms are calculated: $s_{i}=\sum_{l=1}^{n} \left[ a_{l}\cos\left( l\xi_{i} \right)+b_{l}\sin\left( l\xi_{i} \right) \right]$, for each position in the discretization $\Xi_{\mathrm{in}}$. Then, an empirical rectification is done in such a way as to ensure at least one bound energy level: $v_{i}=\frac{7}{10}\cdot\frac{s_{i}-\min_{1\leq i\leq M} \left\{ s_{i} \right\}}{\max_{1\leq i\leq M} \left\{ s_{i}-\min_{1\leq i\leq M} \left\{ s_{i} \right\} \right\}}$. The potential discrete function $\left\{ \xi_{i} \right\}_{1\leq i\leq M}\to\left\{ v_{i} \right\}_{1\leq i\leq M}$ is then obtained by forcing all the values outside the well to be 1. Examples are given in Fig. A3.


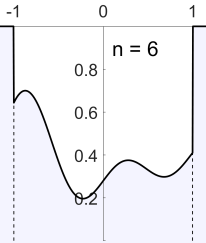

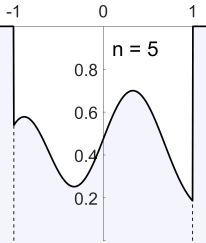

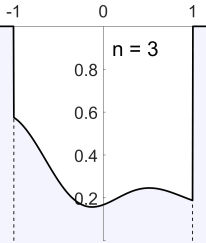

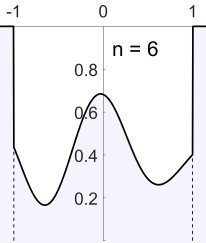

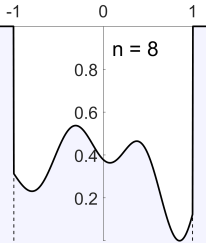

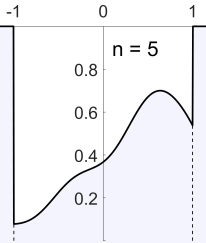

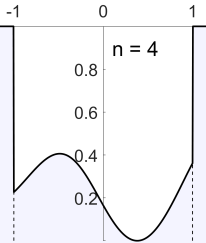

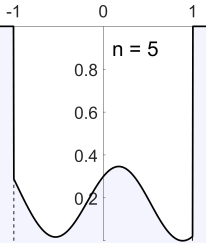

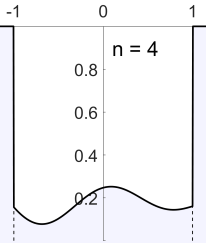

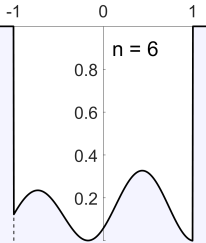

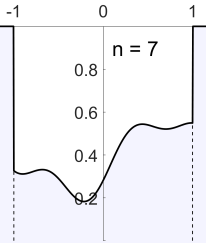

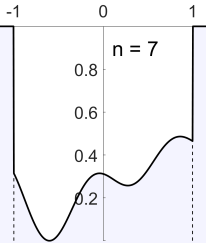

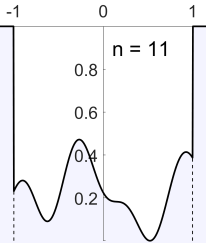

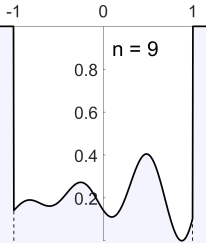

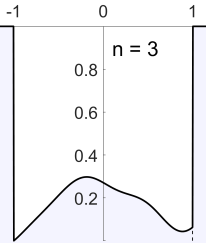

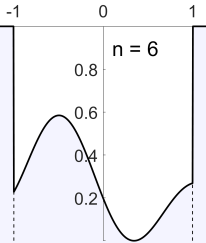

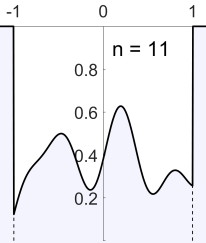

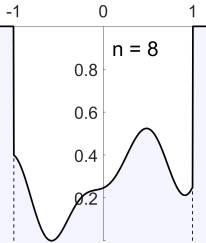


Fig. A3. Several random DS3 potentials.
